# Medical News and Miscellany

**Published:** 1891-07

**Authors:** 


### Medical News and Miscellany.

**Dr. T. J. Tyner and wife** left Austin for Europe via N. Y., June 15, to be absent several months; health and pleasure being the object.

**Errata.**—In our last our compositors made us say, "The necessity for which 'are' clearly recognized.'' Beg pardon, you *know* we know better that that.

---

Dr. W. O. Wilkes, of Waco, the popular Secretary of the Central Texas District Medical Association, is at the N. Y. Polyclinic where he will take a three months course.

---

The University of Louisville medical college had a class of 369 last year, of which 55 were Texans. With improved facilities for instruction the faculty invite the attention of the Texas profession, and solicit a liberal share of patronge for the coming session.—See new announcement.

---

A New St. Louis Journal.—Dr. Bransford Lewis will shortly begin the publication, in St. Louis, of a new medical journal, which, it is predicted, will be "a surprise to the profession.'' We predict it will be an agreeable surprise, if the doctor lets himself loose in his usual happy style. We await number one with anxiety.—See adv.

---

Yellow Fever in Mexico.—State Health Officer Swearingen has been visiting the Mexican frontier posts and instituting extraordinary precautions to prevent invasion of yellow fever into Texas. The report that yellow fever is prevailing at Tampico is denied by Health Officer Harrison, but State Health Officer Swearingen says it is about to assume epidemic form in Vera Cruz. He is confident of being able to keep it out of Texas.

---

The Louisville Medical College has recently purchased a fine site in the central portion of the city and will shortly begin the erection of a $50,000 college building on it; it is also in contemplation to build a complete hospital in connection therewith, all within the college grounds. This college is very popular with the Texas profession, and deservedly so, for it has turned out, as graduates, some of the best practitioners now in the profession, and many of them are in the front ranks. See new announcement; for information address the courteous Secretary, Dr. Geo. M. Warner, Louisville.

---

The Student's Opportunity—A full Course of Lectures Free — DANIEL'S TEXAS MEDICAL JOURNAL will give a ticket to the first course of lectures ($140) in the Medical Department, University of Texas, to the student who will, by the 15th of October, secure one hundred and forty subscribers for the JOUR-

NAL at $2 each--the regular subscription price. The names can be sent in as secured, one or more at a time, and the sender can remit $1.00 for each, retaining the other $1.00, or he can remit in full and have the amount deposited, subject to his order when list is completed.

Here is a rare opportunity, and in the reach of an industrious student. By visiting the several District societies at their sessionsthe required number of subscribers can be easily secured; as the JOURNAL, enlarged and improved, will take readily on sight; has always done so.

---

**The Doctor's Opportunity.**—DANIEL'S TEXAS MEDICAL JOURNAL will give a ticket to a full course of lectures in the St. Louis or New Orleans Post Graduate Medical College to any physician who will secure and send in with the money by October 15, seventy-five subscribers at the regular subscription price of $2. Terms, same as above. Here is a chance to make a $75 post graduate course easily, and to do a good missionary work at the same time.

---

**The Great Medical Temperance Conference** was held at Prohibition Park, Staten Island, N. Y., on the 16th and 17th inst. The paper by Dr. W. A. Morris which created such interest at Waco, was read at the Conference on the 17th, and will appear in the published Transactions. Would that a million copies of it could be struck off and one placed in the hands of every family in the land. The JOURNAL looks for substantial results from this Conference, in the shape of a memorial or petition to Congress. Such memorial, coming from such source, could not be ignored. We suggest that the profession join forces with the W. C. T. U. Association; let them do the praying and let the Doctors abstain from prescribing alcohol, and educate families as to the evils of it.

---

**Our Esteemed Contemporary** of the Richmond *Southern Clinic* (July number) is very severe in denouncing the Judicial Council of the Texas State Medical Association for their action at Waco. We cheerfully give Bro. Bryce credit for good intentions—the defense of the freedom of the medical press; but he was laboring under a misapprehension of facts; had believed too implicitly everything he saw in print; and for that reason has done an injustice to the Association and especially to the Council. No more honorable, conscientious and intelligent men are to be found in the ranks of the Texas profession than those composing the Judicial Council. The published Transactions which will shortly be issued, will give a correct version of the matter,—the minutes of the Secretary,—a synopsis of which was published in this JOURNAL for May.

---

**Quarantine Circular**.—The following circular has been sent out by R. M. Swearingen, M. D., State Health Officer, to the county judges of the State:

"I respectfully invite your attention to the enclosed reprint of the Quarantine Law enacted by the 22nd Legislature. In order to systematize the Quarantine Department, and prepare for prompt and efficient action, when occasion requires such action, it will be necessary for the Honorable County Judges of the State to comply with that part of the law relating to their official duties.

"I therefore earnestly beg that you will at an early date appoint your County Physician; and that you will, as soon thereafter as practicable, be kind enough to give me the name and postoffice address of said physician."

---

**A Private Insane Asylum for Austin.**—There is a movement on foot by Austin physicians to establish a large private insane asylum in Austin. The scheme is materializing and the institution may be looked upon almost as a certainty. It is well known that there is nothing of the kind in Texas—no place where even the victims of vicious habits or of nervous diseases of any kind can receive proper care and treatment, and as the State lunatic asylums do not receive any cases but insanity, and as they must give preference to the pauper insane, unfortunates of the wealthier classes—those able to pay, are compelled to go beyond the State for treatment. Hence this step in one which should comment itself heartily to the people of Texas. It is to be a private enterprise.

---

**The First Annual Meeting** of the U. S. Medical Practitioners' Protective Alliance was held at Baltimore June 11th and 12th. The order was incorporated under the laws of Maryland. Officers for the ensuing year were elected as follows: President, Dr. W. H. Crim, Baltimore, Md.; Vice-President, Dr. W. V. Wilson, New Haven, Conn.; Secretary, Dr. J. F. Davison, Glendola, N. J.; Treasurer, Dr. R. B. Elderdice, McKnightstown, Pa. As usual in meeting for organization, comparatively little work could be done outside the regular routine. An address, setting forth the objects and aims of the society, and dealing with methods whereby physicians might the better enjoy the fruits of their labors, was read by the founder, Dr. J. H. DeWolff. Several other papers on alliance work in general were also read and discussed. Sixteen States were represented, and altogether the meeting was considered a highly successful one. The proceedings will be published in a few weeks.

---

Announcement.—Dr. A. L. Hummel, of 612 Drexel building, Philadelphia, and Mr. Chas. Roome Parmele, of 19 Park Place, New York, have this day, June 24, 1891, entered into a copartnership, operating under the firm name of Hummel & Parmele, the business of which copartnership shall be that of a *Medical Journal Advertising Agency.* Respectfully,

A. L. HUMMEL,
CHAS. ROOME PARMELE.

All contracts made by or with A. L. Hummel or Chas. Roome Parmele individually, since January 1, 1891, are assumed by

HUMMEL & PARMELE.

The JOURNAL endorses the above firm as live, energetic, efficient, and eminently trustworthy. Any business entrusted to them will be attended to in a thoroughly satisfactory manner.

---

The Inter-Continental Medical Congress.—Dr. J. W. Carhart, of Lampasas, the Texas member of the General Committee and Secretary of the I.-C. M. C., writes the JOURNAL: "We are doing a good work and will hold to it. * * * I trust the entire South will make a grand showing in this Congress."

Let it be understood, this General Committee will meet in St. Louis October 14, (1891), for the purpose of organizing the Congress and preparing the program, appointing time and place of meeting, etc. We infer that a representative of each State and district medical society will be appointed on each State committee—the representative of the State on the general committee to be chairman. Dr. F. E. Daniel, the editor of the JOURNAL, has received notice of his appointment on the Auxiliary State Committee to represent the Austin District Medical Society. The selection of Dr. Carhart as Secretary of the Congress is a compliment to Texas, and most worthily bestowed. No more competent and thoroughly conscientious man could have been chosen, or one who would have brought to bear upon the work more zeal and active intelligent interest.

**The Student's Perplexity.**—As is well known, Texas furnishes annually more medical students than any State except Missouri; and has only one medical college. Missouri has fourteen, yet does not manage to grind all the grist of the State. Texas students are now casting around, reading up announcements and college catalogues, preparatory to deciding where they will attend lectures. Where there are so many good colleges, it is really difficult to make a choice; and as so many are represented in our advertising pages, it would be hardly fair in us to discriminate. There is the old Tulane,—whose reputation is like the Rock of Gibraltar, whose clinical facilities are unsurpassed. The younger and very vigorous and enterprising Memphis Medical College, with the glorious "Old Sim" at the helm. The Alabama Medical College, known since *ante bellum* days as one of the highest order of merit, and under the deanship of Dr. Geo. A. Ketchum. The Medical Department University of Nashville, with the popular Drs. Eve and Cain; the Vanderbilt, where Nichol is always a prime favorite with the classes, and where there is always a large Texas contingent. The two Louisville schools—prime favorites and of first class. The two Atlanta Medical Colleges—held in high regard by their *alumni*, some of whom are now ornamenting the Texas profession. The young and healthy College at the foot of Lookout Mountain; the old Jefferson,—the College of Physicians and Surgeons of Baltimore, with Dr. Opie at the head—a high class institution, whose diploma is justly prized. Ditto of the St. Louis, the staunch Missouri Medical, the Marion Sims,—and last but not least, the Gross of Denver—away up in the mountains. *How can* a student choose between them? Read carefully all these advertisements, write to each Dean for catalogue—mentioning that you saw the ad. in DANIEL'S TEXAS MEDICAL JOURNAL,—and then, throw up "heads or tails" to decide where you will go. If we could consistently do so, we would advise you to take one course at each—like sampling, you know—and then shut your eyes and choose at random. This is for those who intend leaving the State; for of course, a large number have already made up their minds to go to the Medical College of our own Texas University, and have already written to Dean West, at Galveston.

---

**The American Society of Microscopists.**—We copy the following extract from a reprint from the *St. Louis Medical and Surgical Journal*, May, 1891:

This association, now in the thirteenth year of its existence will hold its fourteenth annual meeting in Washington, D. C., August 10, and continue in session five days. Its roll of active members contains about three hundred and fifty names. Its membership consists of two distinct classes, viz.: professional men and students of the natural sciences, who use the microscope in their daily avocations as an instrumen t of research, diagnosis, or precision; and amateurs, or those who find pleasure and profit in the revelations of the instrument.

The qualifications for membership are very simple. The applicant must be a respectable person socially, and interested in the use of the microscope.

The dues are $2.00 per annum, and in return the member gets a volume of the Annual Proceedings which costs very nearly this amount. These proceedings are elegantly and profusely illustrated with photo-engravings, autotypes, chromoliths and wood engravings, done in the highest style of art. There is scarcely a subject in the whole range of microscopical work, upon which information may not be found by reference to the indexes of these volumes, and collectively they form a library of microscopy full of invaluable matter to the student and worker.

The museums and libraries, as well as the many other objects of interest of the National Capital and its surroundings, will be open to the visits of the members, and special facilities for seeing them will be accorded.

Special hotel rates will also be secured. An announcement of the railway fares, hotel rates, etc., will be made hereafter.

We invite and urge upon all persons, professional or amateurs, interested in microscopy and not already on the rolls, to send in their applications for membership to the Secretary, Dr. W. H. Seaman, No. 1427 Eleventh street, Washington, D. C. The application should be accompanied by $3.00 which is the initiation fee and one year's dues.

Any further information concerning the Society or the approaching meeting may be obtained on addressing any of the undersigned.

Frank L. James, President, Box 568, St. Louis.

W. H. Seaman, Secretary, No. 1424 Eleventh Street, Washington, D. C.

C. C. Mellor, Treasurer, No. 77 Fifth Ave., Pittsburgh, Pa.

---

**For Sale.**—My practice, with comfortable five room dwelling, office and out-buildings, three acres land, fine orchard, good water; in a fine county, good schools, churches, etc. Tri-weekly mail. No opposition nearer than ten miles. All for $1200, $500 cash down, balance on time to suit purchaser. Failure in health reason for selling. Address Dr. J. C. Baird,
Pidcock Ranch, Texas.

---

**Dr. A. G. Pendlelin**, San Marcos, died July 22d, inst.

